# Supplementary material for: Modelling Skylarks (Alauda arvensis) to Predict Impacts of Changes in Land Management and Policy: Development and Testing of an Agent-Based Model
Source: PLoS One. 2013 Jun 6;8(6):e65803. doi: 10.1371/journal.pone.0065803 (PMC3675089; doi:10.1371/journal.pone.0065803)
Supplement: Supporting Information S4 — The skylark ODdox as a zipped archive. (ZIP) [file pone.0065803.s004.zip › Skylark_ODdox/farm_8h.html]

ALMaSS Skylark ODdox: farm.h File Reference


ALMaSS Skylark ODdox
 2.0


- Main Page
- Related Pages
- Classes
- Files

- File List
- File Members

Classes |
Macros |
Enumerations

farm.h File Reference

**Farm.h This file contains the headers for the Farm class**   
More...

|  |  |
| --- | --- |
| Classes | |
| class | AgroChemIndustryCerealFarm1 |
|  | Inbuilt special purpose farm type. More... |
| class | AgroChemIndustryCerealFarm2 |
|  | Inbuilt special purpose farm type. More... |
| class | AgroChemIndustryCerealFarm3 |
|  | Inbuilt special purpose farm type. More... |
| class | CompareFarmNum |
|  | Function class to compare to farm refs. More... |
| class | ConventionalCattle |
|  | Inbuilt farm type. More... |
| class | ConventionalPig |
|  | Inbuilt farm type. More... |
| class | ConventionalPlant |
|  | Inbuilt farm type. More... |
| class | ConvMarginalJord |
|  | Inbuilt special purpose farm type. More... |
| class | Crop |
|  | The base class for all crops. More... |
| class | Farm |
|  | The base class for all farm types. More... |
| struct | FarmEvent |
|  | A struct to hold the information required to trigger a farm event. More... |
| class | FarmManager |
|  | The Farm Manager class. More... |
| class | NoPesticideBaseFarm |
|  | Inbuilt special purpose farm type. More... |
| class | NoPesticideNoPFarm |
|  | Inbuilt special purpose farm type. More... |
| class | OrganicCattle |
|  | A farm that can have its rotation defined by the user at runtime. More... |
| class | OrganicPig |
|  | A farm that can have its rotation defined by the user at runtime. More... |
| class | OrganicPlant |
|  | A farm that can have its rotation defined by the user at runtime. More... |
| struct | PermCropData |
|  | Used for storing permanent crop data for the farm rotation. More... |
| class | PesticideTrialControl |
|  | Inbuilt special purpose farm type. More... |
| class | PesticideTrialToxicControl |
|  | Inbuilt special purpose farm type. More... |
| class | PesticideTrialTreatment |
|  | Inbuilt special purpose farm type. More... |
| struct | tpct |
|  | Used for storing farmers field size vectors. More... |
| class | UserDefinedFarm |
|  | A farm that can have its rotation defined by the user at runtime. More... |
| class | UserDefinedFarm1 |
| class | UserDefinedFarm10 |
|  | A farm that can have its rotation defined by the user at runtime. More... |
| class | UserDefinedFarm11 |
|  | A farm that can have its rotation defined by the user at runtime. More... |
| class | UserDefinedFarm12 |
|  | A farm that can have its rotation defined by the user at runtime. More... |
| class | UserDefinedFarm13 |
|  | A farm that can have its rotation defined by the user at runtime. More... |
| class | UserDefinedFarm14 |
|  | A farm that can have its rotation defined by the user at runtime. More... |
| class | UserDefinedFarm15 |
|  | A farm that can have its rotation defined by the user at runtime. More... |
| class | UserDefinedFarm16 |
|  | A farm that can have its rotation defined by the user at runtime. More... |
| class | UserDefinedFarm17 |
|  | A farm that can have its rotation defined by the user at runtime. More... |
| class | UserDefinedFarm18 |
|  | A farm that can have its rotation defined by the user at runtime. More... |
| class | UserDefinedFarm19 |
|  | A farm that can have its rotation defined by the user at runtime. More... |
| class | UserDefinedFarm2 |
|  | A farm that can have its rotation defined by the user at runtime. More... |
| class | UserDefinedFarm20 |
|  | A farm that can have its rotation defined by the user at runtime. More... |
| class | UserDefinedFarm21 |
|  | A farm that can have its rotation defined by the user at runtime. More... |
| class | UserDefinedFarm22 |
|  | A farm that can have its rotation defined by the user at runtime. More... |
| class | UserDefinedFarm23 |
|  | A farm that can have its rotation defined by the user at runtime. More... |
| class | UserDefinedFarm24 |
|  | A farm that can have its rotation defined by the user at runtime. More... |
| class | UserDefinedFarm25 |
|  | A farm that can have its rotation defined by the user at runtime. More... |
| class | UserDefinedFarm26 |
|  | A farm that can have its rotation defined by the user at runtime. More... |
| class | UserDefinedFarm27 |
|  | A farm that can have its rotation defined by the user at runtime. More... |
| class | UserDefinedFarm28 |
|  | A farm that can have its rotation defined by the user at runtime. More... |
| class | UserDefinedFarm29 |
|  | A farm that can have its rotation defined by the user at runtime. More... |
| class | UserDefinedFarm3 |
|  | A farm that can have its rotation defined by the user at runtime. More... |
| class | UserDefinedFarm30 |
|  | A farm that can have its rotation defined by the user at runtime. More... |
| class | UserDefinedFarm31 |
|  | A farm that can have its rotation defined by the user at runtime. More... |
| class | UserDefinedFarm32 |
|  | A farm that can have its rotation defined by the user at runtime. More... |
| class | UserDefinedFarm33 |
|  | A farm that can have its rotation defined by the user at runtime. More... |
| class | UserDefinedFarm34 |
|  | A farm that can have its rotation defined by the user at runtime. More... |
| class | UserDefinedFarm35 |
|  | A farm that can have its rotation defined by the user at runtime. More... |
| class | UserDefinedFarm36 |
|  | A farm that can have its rotation defined by the user at runtime. More... |
| class | UserDefinedFarm4 |
|  | A farm that can have its rotation defined by the user at runtime. More... |
| class | UserDefinedFarm5 |
|  | A farm that can have its rotation defined by the user at runtime. More... |
| class | UserDefinedFarm6 |
|  | A farm that can have its rotation defined by the user at runtime. More... |
| class | UserDefinedFarm7 |
|  | A farm that can have its rotation defined by the user at runtime. More... |
| class | UserDefinedFarm8 |
|  | A farm that can have its rotation defined by the user at runtime. More... |
| class | UserDefinedFarm9 |
|  | A farm that can have its rotation defined by the user at runtime. More... |

|  |  |
| --- | --- |
| Macros | |
| #define | PROG\_START   1 |

|  |  |
| --- | --- |
| Enumerations | |
| enum | TTypesOfFarm {     tof\_ConventionalCattle = 0, tof\_ConventionalPig, tof\_ConventionalPlant, tof\_OrganicCattle,     tof\_OrganicPig, tof\_OrganicPlant, tof\_PTrialControl, tof\_PTrialTreatment,     tof\_PTrialToxicControl, tof\_ConvMarginalJord, tof\_AgroChemIndustryCerealFarm1, tof\_AgroChemIndustryCerealFarm2,     tof\_AgroChemIndustryCerealFarm3, tof\_NoPesticideBase, tof\_NoPesticideNoP, tof\_UserDefinedFarm1,     tof\_UserDefinedFarm2, tof\_UserDefinedFarm3, tof\_UserDefinedFarm4, tof\_UserDefinedFarm5,     tof\_UserDefinedFarm6, tof\_UserDefinedFarm7, tof\_UserDefinedFarm8, tof\_UserDefinedFarm9,     tof\_UserDefinedFarm10, tof\_UserDefinedFarm11, tof\_UserDefinedFarm12, tof\_UserDefinedFarm13,     tof\_UserDefinedFarm14, tof\_UserDefinedFarm15, tof\_UserDefinedFarm16, tof\_UserDefinedFarm17,     tof\_UserDefinedFarm18, tof\_UserDefinedFarm19, tof\_UserDefinedFarm20, tof\_UserDefinedFarm21,     tof\_UserDefinedFarm22, tof\_UserDefinedFarm23, tof\_UserDefinedFarm24, tof\_UserDefinedFarm25,     tof\_UserDefinedFarm26, tof\_UserDefinedFarm27, tof\_UserDefinedFarm28, tof\_UserDefinedFarm29,     tof\_UserDefinedFarm30, tof\_UserDefinedFarm31, tof\_UserDefinedFarm32, tof\_UserDefinedFarm33,     tof\_UserDefinedFarm34, tof\_UserDefinedFarm35   } |

---

## Detailed Description

**Farm.h This file contains the headers for the Farm class**

by Frank Nikolaisen & Chris J. Topping   
Version of June 2003   
  
Doxygen formatted comments in July 2008

---

## Macro Definition Documentation

#define PROG\_START   1

Referenced by Farm::HandleEvents(), and Farm::InitiateManagement().

---

## Enumeration Type Documentation

enum TTypesOfFarm

**Enumerator:**
:   |  |  |
    | --- | --- |
    | *tof\_ConventionalCattle* |  |
    | *tof\_ConventionalPig* |  |
    | *tof\_ConventionalPlant* |  |
    | *tof\_OrganicCattle* |  |
    | *tof\_OrganicPig* |  |
    | *tof\_OrganicPlant* |  |
    | *tof\_PTrialControl* |  |
    | *tof\_PTrialTreatment* |  |
    | *tof\_PTrialToxicControl* |  |
    | *tof\_ConvMarginalJord* |  |
    | *tof\_AgroChemIndustryCerealFarm1* |  |
    | *tof\_AgroChemIndustryCerealFarm2* |  |
    | *tof\_AgroChemIndustryCerealFarm3* |  |
    | *tof\_NoPesticideBase* |  |
    | *tof\_NoPesticideNoP* |  |
    | *tof\_UserDefinedFarm1* |  |
    | *tof\_UserDefinedFarm2* |  |
    | *tof\_UserDefinedFarm3* |  |
    | *tof\_UserDefinedFarm4* |  |
    | *tof\_UserDefinedFarm5* |  |
    | *tof\_UserDefinedFarm6* |  |
    | *tof\_UserDefinedFarm7* |  |
    | *tof\_UserDefinedFarm8* |  |
    | *tof\_UserDefinedFarm9* |  |
    | *tof\_UserDefinedFarm10* |  |
    | *tof\_UserDefinedFarm11* |  |
    | *tof\_UserDefinedFarm12* |  |
    | *tof\_UserDefinedFarm13* |  |
    | *tof\_UserDefinedFarm14* |  |
    | *tof\_UserDefinedFarm15* |  |
    | *tof\_UserDefinedFarm16* |  |
    | *tof\_UserDefinedFarm17* |  |
    | *tof\_UserDefinedFarm18* |  |
    | *tof\_UserDefinedFarm19* |  |
    | *tof\_UserDefinedFarm20* |  |
    | *tof\_UserDefinedFarm21* |  |
    | *tof\_UserDefinedFarm22* |  |
    | *tof\_UserDefinedFarm23* |  |
    | *tof\_UserDefinedFarm24* |  |
    | *tof\_UserDefinedFarm25* |  |
    | *tof\_UserDefinedFarm26* |  |
    | *tof\_UserDefinedFarm27* |  |
    | *tof\_UserDefinedFarm28* |  |
    | *tof\_UserDefinedFarm29* |  |
    | *tof\_UserDefinedFarm30* |  |
    | *tof\_UserDefinedFarm31* |  |
    | *tof\_UserDefinedFarm32* |  |
    | *tof\_UserDefinedFarm33* |  |
    | *tof\_UserDefinedFarm34* |  |
    | *tof\_UserDefinedFarm35* |  |

{

tof\_ConventionalCattle=0,

tof\_ConventionalPig,

tof\_ConventionalPlant,

tof\_OrganicCattle,

tof\_OrganicPig,

tof\_OrganicPlant,

tof\_PTrialControl,

tof\_PTrialTreatment,

tof\_PTrialToxicControl,

tof\_ConvMarginalJord,

tof\_AgroChemIndustryCerealFarm1, //10

tof\_AgroChemIndustryCerealFarm2,

tof\_AgroChemIndustryCerealFarm3,

tof\_NoPesticideBase,

tof\_NoPesticideNoP,

tof\_UserDefinedFarm1, //15

tof\_UserDefinedFarm2,

tof\_UserDefinedFarm3,

tof\_UserDefinedFarm4,

tof\_UserDefinedFarm5,

tof\_UserDefinedFarm6, //20

tof\_UserDefinedFarm7,

tof\_UserDefinedFarm8,

tof\_UserDefinedFarm9,

tof\_UserDefinedFarm10,

tof\_UserDefinedFarm11,

tof\_UserDefinedFarm12,

tof\_UserDefinedFarm13,

tof\_UserDefinedFarm14,

tof\_UserDefinedFarm15,

tof\_UserDefinedFarm16,

tof\_UserDefinedFarm17,

tof\_UserDefinedFarm18,

tof\_UserDefinedFarm19,

tof\_UserDefinedFarm20,

tof\_UserDefinedFarm21,

tof\_UserDefinedFarm22,

tof\_UserDefinedFarm23,

tof\_UserDefinedFarm24,

tof\_UserDefinedFarm25,

tof\_UserDefinedFarm26,

tof\_UserDefinedFarm27,

tof\_UserDefinedFarm28,

tof\_UserDefinedFarm29,

tof\_UserDefinedFarm30,

tof\_UserDefinedFarm31,

tof\_UserDefinedFarm32,

tof\_UserDefinedFarm33,

tof\_UserDefinedFarm34,

tof\_UserDefinedFarm35

}TTypesOfFarm;


- CJT
- MSVC
- ALMaSS Working Source
- Landscape
- farm.h
- Generated on Thu Jan 10 2013 13:15:35 for ALMaSS Skylark ODdox by
   1.8.1.1
